# Abstracts and Gleanings

**Published:** 1883-04-20

**Authors:** 


### ABSTRACTS AND GLEANINGS.

**Quackery Years Ago.**—In a French work entitled "The Art of Medicine, or, The True Means of Succeeding in Medicine," published in Paris in 1843, we find the following amusing anecdote, which tends to show that the quackery of to-day is no new thing:

During a journey which Barthez was making in the South of France, he resolved to visit Bordeaux. Arriving in that city, he put up at the Hotel d'Angleterre, which was the rendezvous for all travelers of distinction.

The morning after his arrival, very early, his sleep was broken by a confusion and noise which was going on upon the stairs. It sounded like a crowd of people coming and going, ascending and descending without cessation. Barthez rose in haste, and quietly half opened his door to find out the reason of all this commotion, and to know if they were not patients who wished to consult him; they certainly were patients, but alas! they passed his door as if scorning him, and repaired to an apartment opposite his own, on which was a large placard above the door, bearing the inscription:

"CONSULTATION GRATIS!
MEDICINES ONLY CHARGED FOR."

Barthez closed his door in confusion; during the whole day, and the following one, the mob never ceased.

Lucky confrere! said he to himself; he takes them all, and does not leave even the most trifling consultation to a physician, who, without doubt, is in no sense his inferior (Barthez had good cause to pay himself the tribute). "Who is this man that is in such vogue?" he inquired of the servants in the hotel; the Doctor was only known there by name: his name was Dr. Laurent, and every one repeated it, "It is Dr. Laurent!"

One day Barthez being at the head of the stairs, his unknown confrere emerged from his apartment, muffled in a rich dressing gown and wearing a black velvet cap fringed with gold. He saluted Barthez humbly, who, utterly astonished, suddenly exclaimed: "What, is it you, Laurent?" In fact, it was Laurent, his old servant! "Yes, sir, it is I."

"But how? Since when? Who the deuce made you a Doctor?"

"You, sir; and I owe you my fortune. You remember, without doubt, that when I was in your service, I accompanied you everywhere, in your professional visits, and that you employed me to convey your opinions to your numerous patients. Well, I listened to all that you said, read all that you wrote, and with all this and the help of a few good formulæ, that I had stolen from you, I made a science of my own, which you see has produced me something handsome."

"You astonish me, Laurent; but your success surprises me still more; and I am so much the more astonished that I, who have been here fifteen days, and whose presence in Bordeaux ought to be known, have not had a patient, while you ——— ," he added smilingly; "but what kind of a city is this?"

"It does not differ from others, sir, and fools are plentiful here, as everywhere else. Your assonishment, permit me to tell you, does not become a man of talent like yourself. Answer me: how many sensible people do you suppose there is in a population of 120,000 souls? 500? 1,000? 1,500? I will grant you 2,000. Well, these 2,000 are your property; but the remaining 118,000, who are fools, are mine, and you can look to them for nothing; Hence you need not be surprised at my numerous clientele."

Barthez reddened, said farewell to Laurent, and left Bordeaux the same evening, promising himself, in future, not to have such confidence in his profound wisdom.

This anecdote proves that the supply of quacks will fail the fools, before the supply of fools will fail the quacks.—*Michigan Med. News.*

**Chronic Ovaritis.**—In an article by Dr. Henry Gervis, obstetric physician to St. Thomas' Hospital, London, in the British Medical of the 3d ult, we have in the history of a case reported by him a very succinct narration of the symptoms of Chronic Ovaritis. The principal one is pain in and radiating from one or the other ovarian region, more or less severe; sometimes dull, sometimes acute, sometimes very acute. At first this pain is occasional only, but apt to be pretty regularly provoked by the occurrence of ovulation, getting worse before the catamenia and lessening afterwards. After a time it becomes continuous, but increased by standing or walking; especially is this the case if the ovary be prolapsed or congested; and if it be the left ovary which is affected it is apt to be aggravated before and during action of the bowels. So great is the pain under the latter circumstances that the patient will sometimes defer defecation for one or two weeks, and in one of Dr. Gervis' cases even three weeks were allowed to pass through dread of the pain. In the early stages of ovaritis there is a tendency to menorrhagia, but later on, owing to changes in the tissue, the flow usually becomes scanty and is accompanied by pain (dysmenorrhœa). The pain on pressure over the ovary is of a sickening nature, resembling, indeed, the pain caused by pressure on an inflamed testicle. Among the constitutional symptoms the chief are neurotic disturbances and nervous irritability, the result of nerve exhaustion.

As regards treatment, it must be remembered that uterine trouble usually precedes the ovarian, and the former must be rectified before the latter, *per se*, can be benefitted. The bowels must be maintained in a soluble condition, and much benefit will be derived from injections twice daily of hot water (100° to 110°) and the introduction of pledgets of cotton saturated with glycerin high up in the vagina. All pressure from the clothes must be guarded against, as must also all pressure from accumulation of flatus. Rest in the recumbent posture, within the limits of physiological necessities, must be enjoined, the hips being raised by a pillow. The iodide of potassium for its effect on the inflamed tissue, the bromides of potassium and ammonium for the nervous disturbances.

The local treatment includes the use of iodine or small blisters to the inguinal region, or sedative liniments, such as the liniments of aconite, belladonna, or chloroform. Hot sitz baths, douches, 105°–110°; mercurial vaginal suppositories where it appears possible to promote absorption of exudations, or lessen ovarian congestion; and when the ovary is prolapsed, the introduction of an elastic ring pessary, which supports at once uterus and ovary, often so taking off the dragging and bearing down sensations of which the patient complains, and markedly relieving the dyspareunia so frequently present. A device that Dr. Gervis has made use of sometimes, and with benefit, has been what has been called the postural method of treatment. By getting the patient to kneel for definite periods of fifteen to thirty minutes, two or three times a day, in the genu-pectoral position, the ovaries, unless fixed, will gravitate out of the pelvis, and so lose some of their congestion. And these approaches to a healthy condition, repeated frequently, lead, it is believed, to the tendency toward health becoming permanent. At all events this position very frequently has the effect of distinctly relieving pain.

One other point worthy of attention is the question of utero-gestation; and the cognate one of the permissibility of conjugal relations in the married. Briefly, sexual excitement being obviously undesirable, intercourse must be within the most restrained limits, when from circumstances, complete abstinence is unattainable. In some stages of ovaritis, while ovulation proceeds, conception is of course possible; and there is also no doubt that gestation has, in many cases, been distinctly beneficial to cases of chronic ovaritis. The explanation being that during gestation the ovaries are having a complete physiological rest; and also that being lifted out of the pelvis by the uterus, as it rises in the abdomen, they are removed from much of the pressure and weight to which, while in the pelvis, they are subjected. But in spite of all such precautions and treatment, a certain number of these cases drift into the third class, the class in which no remedy short of a nearly persistent narcotism appears to give any relief. The patient is always in pain; pain spoken of by some as a burning, scorching pain; by others as a wearing, unendurable pain; by others as "torture;" a pain from which nothing but the stronger sedative, or hypodermic injections of morphia, or large doses of brandy, give any freedom. For the relief of this class little but surgical treatment remains, apart from the perpetual administration of sedatives.—*Medical Age.*

**Sodium Sulpho-Carbolate in Vomiting.**—Dr. Philip McCall (British Medical Journal, December 16th, 1882), has found that sulpho-carbolate of sodium is of great benefit in the vomiting of pregnancy in seven-grain doses in a half ounce of water. In one case of sea-sickness it had a good effect.—*American Med. Weekly.*

**Rape During Hypnotic Slumber.**—Dr. Ladome, of Neuchatel, publishes in the Annales de Hygiene Publique, a very interesting report upon a medico-legal question. At the close of some entertainments in mesmerism given at his canton, the young people were possessed by a magnetic fever. One of the consequences of this was that a young girl became enciente, and declared that being alone Christmas eve with a young man, who was in the habit of magnetizing her, he had violated her after having put her to sleep. The affair was submitted to a Justice, and Dr. Ladome appointed to make a medico-legal report bearing principally upon the following questions:

(1). The story of the plaintiff, ought it to be considered probable as a whole?

(2). Could coition take place without her consciousness of the fact at the time?

(3). Was her will so paralyzed that she was unable to offer any resistance?

(4). Is conception possible in a state of absolute insensibility?

Dr Ladome remarks that this is a new question in legal medicine, there existing but four cases in medical literature, dating from 1858.

Making observations on the possibility of simulation, M. M. Devergie, Tardieu and Bronardel arrived at the conclusion that a girl could be violated while her will was abolished in a nervous or hypnotic sleep.

Passing in review her story that she was awakened at a certain time, and again put to sleep without being able to resist, Dr. Ladome concludes there is nothing in this contradictory to the phenomena of hypnotic slumber. That coition could have taken place without her knowledge, is not to be doubted, as she could have been rendered absolutely insensible. The third question is more difficult: Was her will so completely paralyzed that she could offer no resistance? It may, however, be answered affirmatively; for in furnishing his subject with an appropriate theme of hallucination, a skillful operator could, with certain persons, provoke actions en rapport with the dream developing in their over excited imaginations. It would seem then that the operator has really the power to direct according to his inclination the will of the subject, while in reality he directs only a hallucination, but the subject is none the less at his mercy. It remains to know if conception is possible when the woman is in a state of complete insensibility. Every author admits that the sole condition necessary to fecundation, is the meeting of the semen and the ovum in the female.

The magnetiser proved an alibi, and the case was dismissed.—*Obstetric Gazette.*

**A Recent Decision** holds that if a surgeon recovers his fee in a suit for the value of his services, no subsequent suit can be brought for malpractice, this being settled by the results of suit for services.—*Detroit Lancet.*

**Treatment of Eczema of the Genitalia, Pruritus and Leucorrhœa.**—In cases of eczema, in which glyceroles and unguents have failed, the following formula has been successful :

℞ Chlorate of potassium .................... 30 grains,
Wine of opium .......................... 50 grains,
Pure water, ............................ 1 quart.

Applied to the parts by linen compresses covered with oil silk. If there is much inflammation, precede this with warm hip baths and cataplasms sprinkled with powdered carbonate of lime. In obstinate pruritus, associated with leucorrhœa, a tablespoonful of mixture of equal parts of tincture of iodine and iodide of potassium, in a quart of warm tar water (tar water holding the iodine in solution) used daily, night and morning, removes the pruritus and ameliorates the leucorrhœa. In fetid leucorrhœa two or three tablespoonfuls (in a quart of warm water morning and evening, as an injection) of the following formula will be found useful :

℞ Chlorate of potassium .................... 13 grams,
Wine of opium .......................... 10 grams,
Tar water .............................. 300 grams.

Or—

℞ White vinegar (or wine) ................. 300 grams,
Tinct. eucalyptus ....................... 45 grams,
Acid salicylic, ......................... 1 gram,
Salicylate of soda ...................... 20 grams.

One to five teaspoonfuls in a quart of warm water as an injection two or three times a day.—*Obstetric Gazette.*

**Sponge-Grafting.**—Dr. W. H. Thorndike and Dr. C. D. Homans, of the Boston City Hospital (Medical Record, October 7, 1882), reports four cases of this treatment in different classes of wounds, the only four in which it has been tried at that institution; but the results have not been so remarkably satisfactory as some of those reported in the English journals. They conclude, however, that the treatment is useful in a certain limited class of cases, such as deep, circumscribed ulcers of the leg, whieh are often followed by adherent cicatrices which readily break down. In these cases the sponge forms a trellis-work through which the granulation springs up from the bottom of the wound, thus preventing the contraction from the edges. The sponges used in grafting should be perfectly clean and thoroughly carbolized. They should then be soaked in dilute acid until they become quite friable, in order that they may be absorbed more rapidly.—*N. Y. Medical Journal.*

**Iodide of Potassium in Frontal Headache.**—Dr. Haley, in Australian Medical Journal, claims that minimum doses of iodide of potassium is of great service in frontal headache. A two-grain dose dissolved in half a wineglass of water will often cure a dull headache which is situated over the eyebrow. The action of the drug is quite rapid.—*Med. Summary.*

**Dislocations of the Thigh Reduced by New Methods of Manipulation.**—In cases where reduction of the femur by manipulation in the usual way, with the aid of anæsthetics, has failed, or is inapplicable, and as a substitute, in many cases, for anæsthesia, and mechanical power, Mr. Kelley (Dublin Journal of Medical Science) proposes the following methods:

FOR POSTERIOR DISLOCATIONS.

The patient is laid prostrate on the floor. Three strong screw-hooks are inserted into the flooring close to the perineum and each ilium of the patient, and to these hooks he is secured by strong bandages or rope. The injured thigh is flexed at right angles to the patient's body; the foot and lower extremity of the tibia are placed against the perineum of the surgeon, who, bending forward, with the knees slightly flexed, passes his forearms behind the patient's knee and grasps his own elbows. Reduction is now accomplished by drawing the femur upwards; but circumduction may also be practiced; the surgeon, stepping backwards, then extends the limb, and lays it by the side of its fellow. In sciatic dislocations, in order to liberate the head of the bone from the foramen, a bandage may be passed around the thigh, close to the trochanter, by which an assistant may make traction.

FOR ANTERIOR DISLOCATIONS.

The patient is placed upon a table of such elevation as to have his pelvis nearly as high as the trochanter of the surgeon. A bandage around the pelvis, and secured to the side of the table farthest from the dislocation, affords counter-extension. The surgeon, with his face directed towards the dislocated joint, and standing on its inner side, with his trochanter pressed against the femur, now bends the leg behind his back, and grasps the ankle with the corresponding hand. Reduction is effected by rotating or turning his body partially away from the patient, thus making traction on the femur in the most favorable direction, and at the same time pressing the head towards the acetabulum with the disengaged hand.—*Med. Times.*

**Medical Education of Women in Russia.**—The authorities in Russia are evidently not in favor of the medical education of women. The lecture courses at St. Petersburg have been closed by order of the Emperor, after an existence of ten years. The government has deprived the institution of its buildings, and maintains that the institution had not the means to carry it on properly. Subscriptions were promised, but every obstacle was thrown in the way of their collection. The experiment of female practitioners has evidently been a failure in that country at least.—*Lancet.*

**In the ruins** of Pompeii has been lately discovered a quadrivalve speculum, exquisitely proportioned, with a movement unsurpassed by the most perfect of modern instruments.—*Detroit Lancet.*

**Perils of Medical Practice.**—Our English exchanges give graphic accounts of a sad case which recently transpired in England, and which illustrates some of the dangers to which medical practitioners are subject.

A Dr. Edwards, of Hounslow, was accused by a female patient of an assault on her chastity, and a demand made on him for money as a condition of reticence on the part of woman. In his hour of trial, Dr. Edwards naturally consulted his partner, who, instead of coming to his support seized upon the opportunity to dissolve their business relations. Thus attacked and suspected by the one who should have stood by him, at least until his guilt was established, poor Edwards, doubtless losing all hope of establishing his innocence, gave up the fight, and by means of a dose of prussic acid, placed an impassable barrier between him and his persecutors. He left behind him a note written on the very brink of eternity, asservating his innocence of the charge brought against him by "the morbid imagination of a licentious-minded, hysterical woman," and praying for a "blessing on his wife, his little boys, and his mother." The woman, struck by remorse at the suicide of her victim, made a written retraction of the charge, with every expression of regret.

Popular sympathy for the family of the deceased is deep, and everything possible has been done to mitigate the pain of their misfortune, while the indignation against Dr. Whitemarsh, the partner, took shape in the storming of his house by a mob, who would also have lynched him but for the protection of a posse of forty constables.

On the heels of the above case comes the following: The prosecutrix, a young girl, came to Dr. Sparrow, complaining of morning sickness, headache, and total suppression of menses for the past five months. Suspecting from her general appearance that the patient was pregnant, Dr. Sparrow refused to give any emmenagogue medicine (which she urged him to do), until satisfied that she was not *encinte.* To ascertain this, he—with her full consent—made an examination which fully confirmed his previous suspicions. The patient then left, and the next thing the Doctor heard of the matter was a summons for assault. Fortunately, he was able to produce the evidence of three visitors and three servants, who were all within ear-shot at the time, yet they heard no outcry.

The magistrates, convinced that the whole affair was "a plot," at once dismissed the charge, and no doubt the lapse of a few months will confirm Dr. Sparrow's diagnosis and his perfect innocence. We are well pleased to observe that Dr. Sparrow's medical brethren, having satisfied themselves of his freedom from guilt, stood by him in his adversity.

In provincial practice it is obviously impossible for a practitioner to have, at all times, the safeguard of witnesses when it becomes necessary to make gynecological examinations, and it is daily becoming more manifest that charges of immorality against medical men ought to be regarded with the utmost suspicion.

These sad cases have their lesson. While we have a feeling akin to contempt for a man who suicides to avoid trial and sorrow, particularly when he has within him the consciousness of innocence, our sympathies are touched by the salient features of the case. Physicians are constantly liable to similar charges to these, and we have known of instances in which in spite of innocence, they have paid money to avoid scandal. The only safeguard is that suggested by our contemporaries, viz: that physical examinations of women should be made only in the presence of a third and reliable party. The woman who objects to this should be sent elsewhere.—*Medical Age.*

**The Bacillus of Whooping-Cough.**—An interesting paper, pertaining to the pathology of whooping-cough, was lately read before the Medical Society of London, by Mr. Dolan, F.R.S., in which views were advocated which were put forth by Poulet, in 1867; by Letzerich, in 1873; and still later, by Tschamer. Mr. Dolan has repeated the experiments of these former investigators, and with successful results.

Poulet, in 1867, found certain bacteria of a peculiar kind in the sputa of patients affected with pertussis; Letzerich commenced a series of investigations a few years later. The latter found constantly present in the sputum of pertussoid patients a bacterium belonging to the genus Ustiligo, Tul.; with this micrococcus he inoculated the tracheal mucous membrane of tracheotomized rabbits and noted the results. He invariably produced a spasmodic catarrhal affection resembling whooping-cough, and he observed that the bacteria do not penetrate the epithelium, but live on the surface of the mucous membrane, to the detriment of the latter.

Tschamer, of Gratz, working in the same department of micropathology, has lately found, in the expectoration of pertussis, a microphyte, which he identifies with a black mould which develops on orange-peel. This he thinks he has proved by different cultures. Satisfied of the identity, he took some of the black powder which constitutes the mould of orange-peel and experimented with it on himself, inhaling the powder as deeply as he could. At first no effect was observed, but after eight days he began to have convulsive fits of coughing, and expectorated the fungus in abundance.

He explains the phenomena of whooping-cough in this way. After an incubation of seven days, these microphytes determine an irritation of the bronchi which induces catarrh and spasmodic cough; then, as the irritation increases, the expectoration becomes more abundant and eliminates the fungoid organisms.

Dolan, in repeated experiments, found that by inoculating rabbits with the sputa of whooping-cough patients, he not only induced a catarrhal spasmodic affection, but the death of the animal generally ensued. Inoculation with the blood of such patients was without effect. This certainly seems to confirm the conclusions of Letzerich, that the *materies morbi*—be it a bacillus, or be it what it may—lives on the surface of the epithelium, and does not get into the blood.

Dolan does not claim to have arrived at certain knowledge respecting the special bacteroid which causes pertussis.

The theory, then, is a simple one, that whooping-cough, like other contagious diseases, is the product of germs, which, given off in the breath of pertussoid patients, are inhaled by persons of proper susceptibility, and set up irritation of the respiratory epithelium; the result is the vascula and nervous disturbances, and other phenomena which characterize whooping cough. The severe constitutional disturbance which sometimes attends the disease is a secondary effect.

Mr. Dolan suggests nothing new with regard to the treatment of this affection (which must be largely directed to the palliation of symptoms); but adds that if the dependence of pertussis upon a specific virus, be the true explanation of its pathology, the lines on which its rational treatment and prophylaxis are to be pursued become clearer and more hopeful.—*N. Y. Med. Record.*

**Veratrum and Arsenic in the Treatment of Phthisis.**—Readers of the Journal will remember that Prof. Howe strongly recommended these remedies in pulmonary consumption, claiming cures of well marked cases from their continued use. His statements were disputed on general principles, *i. e.*, that veratrum was neither expectorant, stimulant nor tonic, but a sedative or depressant, and arsenic—well, arsenic was the next door neighbor to the devil. Then the treatment did not stand the test of experiment, cases in the advanced stages being selected for the trial. Physicians have a habit of testing new treatment upon patients who have run the gauntlet of medicine.

But there is something in this method, if it is tested fairly in the early stage of the disease, and we can account for the curative action of both remedies in a rational way. The marked symptoms of a tuberculosis are: a frequent pulse and a high temperature. So long as the pulse remains frequent and the temperature high, the disease progresses; when they come down the disease goes slowly or stands still. Veratrum lessens the frequency of the pulse, reduces the temperature, and instead of being depressant, it improves every function. Arsenic in small doses is a *vital stimulant*, improving the appetite, digestion, blood-making and nutrition.—*Eclectic Med. Journal.*

**Reliquet's Injection for Chronic Cystitis.**—According to L'Union Medicale, this consists of crystalized phenic acid, dissolved, with the aid of a sufficient amount of alcohol, in from one thousand to two thousand parts of diluted water. Properly prepared, it is said to give rise to no pain, but to act as an astringent, modifying the denuded surfaces and hindering absorption. After having washed the bladder out with water of the temperature of the body, the solution is injected very slowly, until the bladder is well distended, so that the liquid comes in contact with every portion of its interior. The injection is then allowed to flow out, and the organ is again washed out with warm water.—*N. Y. Med. Record.*

**The Value of Cannabis Indica in Checking Epistaxis.**—Dr. W. G. Maxwell, ot Still Pond, Maryland, sends the following communication:

"The recent attack of epistaxis from which Governor Hamilton (of this State, Maryland) suffered, prompts me to call the attention of the medical profession to the above named drug, which has acted like magic in checking epistaxis during the seven years I have been using it.

I have had nine cases of profuse epistaxis (where plugging the nares seemed to be the only alternative) that were checked by Indian hemp in from three to twenty minutes. Nor was there a recurrence of hemorrhage in a single case.

I have prescribed it for a number of other persons subject to bleeding at the nose, who derived the same benefit from it.

I use the tincture in ten to twenty drop doses, repeated every five to ten minutes. The largest quantity given was fifty drops in three doses, to a gentleman who had been bleeding ten hours; the hemorrhage ceased in twenty minutes after the first dose was administered.

The cannabis indica was used alone in these cases, there being no other internal or local treatment.—*Peoria Med. Monthly.*

**Chamomile in Infantile Diarrhœa.**—Dr. Christopher Elliott, Physician to the British Hospital for Sick Children (Practitioner, December, 1882), endorses Ringer's claim for the great value of infusion of chamomile in infantile diarrhœa connected with dentition, and in which the stools are many in number, green in color, or are slimy and streaked with blood, and accompanied by pain and cramp. He gives ʒss—ʒj of the infusion to a child under one year, and double the quantity to a child over that age, giving it three times a day or oftener, according to the severity of the attack. He explains the rationale of this treatment by the power which chamomile flowers possess of subduing reflex excitability, a power residing in the volatile oil contained in them. Grisan was unable to tetanize, by means of strychnia, a decapitated frog which had been fortified with a dose of chamomile oil. and *vice versa* when reflex excitability had been artificially produced by means of strychnia, it could be calmed again by chamomile oil.—*Medical Age*

**Aconite and Belladonna in Erysipelas.**—A writer in Medical and Surgical Reporter says: I wish to say a few words in regard to the treatment of that disease. For the last three years I have been treating it with aconite and belladonna, and with much better results than with other methods of treatment, either locally or constitutionally. I have found it superior to iron and quinine, or any treatment that I have used or seen used. The inflammation will begin to subside in from twelve to twenty-four hours, patient will feel more comfortable, and will go on to a speedy recovery. I have used it in cases of all ages, running from fifteen months to fifty years, and with like results in all. I generally give one drop of the tincture of aconite root, and two drops of the fluid extract of belladonna every hour, according to age. I very often direct a mixture of acid carbol., tinct. iodinæ and glycerinæ to be used locally. This is unnecessary as to cure, but it adds to the comfort of the patient. I have also found belladonna a very useful drug in the treatment of various skin diseases. I very frequently combine the fl. ext. and liq. pot., arsenit., and cure cases that have resisted other treatment.

**The Bacilli of Tubercle only Fat-Crystals.**—At a meeting of the Pathological Society held at the Charity Hospital, New Orleans, on November 20th, Dr. H. D. Schmidt, President of the Society, made a demonstration with the aid of the microscope to test the alleged discovery by Prof. Koch, of Berlin, of the bacilli of tuberculosis, or the germs of consumption. Dr. Schmidt has devoted much time to the observation of the bacilli of tuberculosis, and while engaged in his researches the discovery of Dr. Koch was announced to the world. This only encouraged Dr. Schmidt to renewed efforts, and the result was that he ascertained, beyond doubt, he claims, that Dr. Koch's germs were pseudo-bacilli, that is, fatty crystals, and not true bacilli. This result, it was stated, was obtained by long and arduous labor, in which the diseased organs of persons that had been afflicted with pulmonary consumption were minutely and carefully examined. Dr. Schmidt succeeded in finding the crystals, which were similar in appearance to those discovered by Dr. Koch, and evidently the same. To determine their nature, Dr. Schmidt subjected the crystals to the action of boiling ether, when they disappeared, proving that they were not germs or organisms, otherwise they would have remained unaffected by the ether.—*Med. News.*

**Gelseminum Sempervirens in Tetanus.**—Dr. J. B. Read, Tuskaloosa, Alabama, (British Medical Journal. December 23d, 1882), has reported a case of tetanus cured by forty minum doses of the fluid extract of gelseminum sempervirens every two hours. Tetanus, it must be confessed, is a self-limited disease, and certain cases have recently been reported in which sustenance of the patient's strength answered every indication.—*American Medical Weekly.*

**A New Vegetable Styptic.**—The Lancet quotes from the Neuefreie Presse, a statement concerning a plant discovered during the French expedition to Mexico which, when chewed or crushed, exceeds in its styptic action all other substances yet known. Among the natives it is given a name that may be rendered *fowlwort* (*Tradescantia erecta*, Jacq.). It has been acclimatized at Versailles.—*N. Y. Med. Journal.*

**Cholera** is said to have been prevailing at Mecca since the 24th of October, and in Cochin China since the close of the summer.—*N. Y. Med. Journal.*

**Whooping-Cough.**—In a paper read before the London Medical Society, Dr. Dolan (Lancet, October 21st, 1882) dissented from the view of Guineau de Mussy that the affection was produced by swollen bronchial glands pressing on the vagus. This was disproved by two facts: first, the glands could be swollen without causing whooping-cough; second, they were not always swollen in the disease. Dr. Dolan adopts the old view that pertussis is caused by a fungus; Poulet has found bacteria in the sputum of pertussis, and by inoculating rabbits with this sputum Letzerich has given them pertussis; Dolan has found that inoculations with bacteria of pertussis has no effect on animals; inoculation with sputa causes death by pertussis. As might be expected, no pathognomonic pathological lesions were to be found in pertussis. Death usually resulted from complications. Glycosuria was present in fourteen out of fifty of Dolan's. Isolation was the first indication, treatment of complications the next. Antiseptic treatment of the disease itself was likely to have good results.—*Amc. Med. Weekly.*

**Calomel in Diphtheria.**—Dr. Chas. S. Miller reports (Southern Practitioner) a case of diphtheria in which the breathihg was very much embarrassed by the membrane. Calomel in 10-grain doses every hour, until twelve doses were given, was followed by prompt recovery, the membrane being thrown off and showing no tendency to re-form. Neither catharsis nor emesis followed these apparently heroic doses. The case seems strongly corroborative of the claims made by Dr. Reiter in a recent number of Squibb's Ephemeris. Dr. Reiter, however, recommended the calomel in the same sized doses before the membrane appeared, and to prevent its formation, having little or no faith in this treatment after the patch had formed. We should be very much pleased to receive any report on the use of calomel as above. Dr. Reiter's claims for the drug employed in this manner are too positive to be allowed to pass without subjecting it to a trial.—*Med. Age.*

**Pop-corn a Remedy for Vomiting of Pregnancy.**—Dr. Wallace, in Medical and Surgical Reporter, having failed with all the usual methods, in an obstinate case, says:

"Some pop-corn was immediately prepared, a little salt sprinkled thereon, and of which she ate a large saucerful. At my call next day, found she had passed a quiet night, without nausea or vomiting, and had eaten very freely of the pop-corn. She has had no further trouble. January 25, as I was passing her house, I saw her at the door. She informed me that since my first visit she has vomited but once, and then but slightly. Sometimes has slight nausea, which is always relieved by eating some pop-corn.

The remedy is so simple, so easily procured, so utterly harmless, and with me has always proved so efficacious, that I publish this in the Reporter, confident that my professional brethren will find it singularly efficacious in the relief of this most distressing disorder.

**The Cure of Saccharine Diabetes.**—In a paper by Dr. G. Felizet, read before the Academy of Sciences August 14, says the Journal d'Hygiene, the author claims to have discovered a remedy for a disease usually regarded as incurable—saccharine diabetes. The author states that he has succeeded in putting an end to glycosuria artificially produced in animals, and that the medicine that suppresses that artificial glycosuria wiil likewise cure diabetes in a few weeks or months. There exists, says he, a bond of union between artificial glycosuria, intermittent diabetes and confirmed diabetes, and that bond is irritation of the rachidian bulb. It is not, then, in masking the disease by submission to the severities of a regime exempt from bread, feculents, sugar, etc., that we succeed in curing it, but by tapping the very source of the production of sugar, that is to say, by suppressing the irritation of the bulb. Bromide of potassium, by the elective action of sedation that it exerts on the functions of the bulb, suppresses the effects of such irritation with a rapidity that is often surprising, and, in large and repeated doses, cures the diabetes.—*Journal of Health.*

**A Bar to an Action for Malpractice.**—An interesting decision to physicians and surgeons was recently rendered in the courts of one of the Western States.

The services of a surgeon in a particular case were not regarded as satisfactory by the patient, and his bill was refused payment, and, in a suit brought to recover the amount of the bill, the patient defended on the ground that the services were of no value. The decision of the court was in favor of the surgeon, and thereupon the patient brought a direct suit against him, claiming damages for malpractice. Upon the trial this suit was dismissed, the court holding that the question of malpractice had been in effect adjudicated in the former suit under the issue that the services were of no value.

This decision is in accord with one rendered in this State in 1878, and it is no doubt the general rule that the question of malpractice is set at rest by a favorable decision in an action to recover the value of services.—*N. Y. Med. Record.*

**Chest Development by Exercise.**—Dr. Catheart, Lecturer on Anatomy, in the Edinburgh School of Medicine, in a lecture delivered at the Edinburgh Health Society, a short time since, gave, as to the effects of exercise in expanding the chest, some striking facts which related to a school where physical exercise had been systematically carried out. The effect of regular exercise was shown as follows: New boys, aged fourteen, average measurement, 29-3; at fifteen, 30-16; at sixteen, 32-0; at seventeen, 32-6; and at eighteen, 32-5; while former boys measured respectively, 30-6, 32·1, 34-2, 35-8 and 36-8.—*Med. and Surg. Rep.*

**Petroleum**, says the Detroit Clinic, is a rapid solvent of the false membranes, and must possess distinct advantages under such circumstances. This fact, coupled with its antiseptic property, renders it a promising remedy, although its odor always renders its use disagreeable.

